# Impact of Oxidative Stress on Male Reproduction in Domestic and Wild Animals

**DOI:** 10.3390/antiox10071154

**Published:** 2021-07-20

**Authors:** Eliana Pintus, José Luis Ros-Santaella

**Affiliations:** Department of Veterinary Sciences, Faculty of Agrobiology, Food and Natural Resources, Czech University of Life Sciences Prague, Kamýcká 129, 165 00 Prague, Czech Republic; ros-santaella@ftz.czu.cz

**Keywords:** antioxidant, infertility, livestock, ROS, semen, sperm dysfunction, wildlife

## Abstract

Oxidative stress occurs when the levels of reactive oxygen species (ROS) overcome the antioxidant defenses of the organism, jeopardizing several biological functions, including reproduction. In the male reproductive system, oxidative stress not only impairs sperm fertility but also compromises offspring health and survival, inducing oxidative damage to lipids, proteins and nucleic acids. Although a clear link between oxidative stress and male fertility disorders has been demonstrated in humans and laboratory rodents, little information is available about the implications of impaired redox homeostasis in the male fertility of domestic and wild animals. Therefore, this review aims to provide an update regarding the intrinsic and extrinsic factors that are associated with oxidative stress in the male reproductive system and their impact on the reproductive performance of domestic and wild animals. The most recent strategies for palliating the detrimental effects of oxidative stress on male fertility are reviewed together with their potential economic and ecological implications in the livestock industry and biodiversity conservation.

## 1. Introduction

Oxidative stress is defined as a failure in the regulation of redox signaling due either to the overproduction of reactive oxygen species (ROS) or the exhaustion of regulatory antioxidant mechanisms [1]. In addition to this biochemical definition, Costantini [2] has recently proposed that oxidative stress can be biologically defined as any change in one of the molecular components of the redox system that influences the fitness outcomes. The biochemical oxidative stress might not necessarily translate into biological oxidative stress if the signaling activity of ROS stimulates the mechanisms that protect the organism [2]. The intensity and duration of oxidative stress may indeed influence the cell’s fate, ranging from adaptive responses to apoptosis or necrosis [3].

Among biological functions, reproduction is a typically energy-demanding activity that incurs elevated metabolic rate, which is predicted to result in greater ROS generation [4]. In the male reproductive system, testes typically produce high levels of ROS and are more vulnerable to oxidative damage compared to other tissues, in spite of their greater antioxidant capacity [5]. Additionally, spermatozoa themselves are very susceptible to the attack by ROS given their limited antioxidant defense and the high content of polyunsaturated fatty acids (PUFAs) in their membranes, whose composition varies both between [6] and within species [7]. Due to the presence of double bonds in their molecules, PUFAs are very sensitive to lipid peroxidation, which is an autocatalytic and self-propagating reaction that results in the loss of membrane functionality and integrity [8]. Sensitivity to lipid peroxidation also varies across sperm regions, probably as a consequence of their different lipid composition. For instance, frozen/thawed bovine (*Bos taurus*) spermatozoa show more intense lipid peroxidation in the sperm flagellum than in the head [9], probably as a consequence of greater PUFA content in the former [10]. On the other hand, the higher percentage of PUFAs in the plasma membrane is associated with reduced levels of lipid peroxidation and higher sperm quality in freshly ejaculated equine (*Equus caballus*) spermatozoa [7]. The type and content of PUFAs in the sperm membrane also contribute to explain sperm cryotolerance across species, being, for instance, positively correlated with the levels of docosahexaenoic acid in marsupials [6]. Besides their deleterious effects, a small amount of ROS (typically hydrogen peroxide H_2_O_2_, superoxide anion O_2_^•−^, and nitric oxide ^•^NO) plays a key physiological role in the fertilization process by promoting sperm capacitation, acrosome reaction, and sperm-zona pellucida binding (in horses: [11]; in cattle: [12]; in buffalos, *Bubalus bubalis*: [13]; in pigs, *Sus scrofa*: [14]). In boar spermatozoa, Betarelli et al. [15] recently found that sperm capacitation is associated with disruption of disulfide bonds and increased intracellular ROS levels, which were prevented by the addition of glutathione (GSH) in the medium. However, above physiological levels, ROS induce oxidative damage to sperm proteins, lipids, and nucleic acids that finally results in sperm death. It is important to note that the impact of oxidative stress on sperm function may vary within species, depending on the type and concentration of ROS, as it has been described both in domestic (pigs: [16]; dogs, *Canis lupus familiaris*: [17]) and in non-domestic (red deer, *Cervus elaphus*: [18]) animals. By comparing three ROS-generating systems (i.e., Fe^2+^/ascorbate, H_2_O_2_, and hypoxanthine/xanthine oxidase), Martínez-Pastor et al. [18] found that H_2_O_2_ is the most cytotoxic ROS for cryopreserved red deer epididymal spermatozoa. Similarly, in dogs, epididymal and seminal plasma-free ejaculated spermatozoa are highly susceptible to H_2_O_2_ and ^•^OH, but relatively more resistant to O_2_^•−^ [17,19]. Within species, the same type of ROS may also have a different impact on sperm function depending on its concentration: for instance, 300 μM H_2_O_2_ induces a significant decrease in boar sperm motility after 30 min of incubation, whereas it had no effect on mitochondrial membrane potential even after 120 min of sperm incubation [16]. Surprisingly, in stallions, 50 μM H_2_O_2_ significantly reduces sperm DNA fragmentation, while it does not affect any other parameter when compared to the control group [20]. Overall, motility is considered to be the most sensitive sperm trait to the effects of oxidative stress ([21]; see also [22]).

From an ecological perspective, oxidative stress has been proposed as the mechanism that may explain the trade-offs between reproduction and self-maintenance, as it has been predicted by the life-history theory [23]. However, empirical tests of this theory have been mainly focused on females and often yielded equivocal support [4]. The discrepancy found among studies might be due to a wide variety of intrinsic (e.g., species, sex, age, reproductive strategies, short- or long-lived species) and extrinsic (e.g., season, pollution, experimental conditions, laboratory assays) factors, which may influence the oxidative status of the individual. In zebra finches (*Taeniopygia guttata*), for instance, males seem to be more vulnerable than females to the oxidative stress caused by increased breeding effort [23], which might be affected by their early developmental conditions [24]. In contrast, in the northern elephant seals (*Mirounga angustirostris*), both sexes show increased oxidative stress during the breeding season, but they differ in the type of oxidative damage: males show increased lipid and DNA damage, while females show increased protein damage [25]. In males, the oxidative cost of current reproduction may also translate into a reduced survival rate (e.g., in the Seychelles warblers, *Acrocephalus sechellensis*; [26]). In this way, in the tarantula (*Brachypelma albopilosa*), the increased oxidative damages of males might contribute to explaining their shorter life expectancy (1–2 years) compared to that of females (up to 20 years) [27].

Although a clear link between oxidative stress and male fertility disorders has been demonstrated in humans and laboratory animals [28], less information is available about the implications of this condition in the male fertility of domestic and wild animals. The purpose of this review is to illustrate the variety and complexity of intrinsic and extrinsic factors that are associated with oxidative stress in the male reproductive system and their impact on the male fertility of domestic and wild animals.

## 2. Intrinsic Factors Associated with Oxidative Stress in the Male Reproductive System

Several intrinsic factors are associated with oxidative stress in the male reproductive system both at the cellular and individual levels (Table 1). At the cellular level, sperm metabolism and the incidence of immature, abnormal, or dead spermatozoa are associated with ROS overproduction. Moreover, leukocyte activation following inflammation or infection has been shown to induce oxidative stress in spermatozoa. In general, the male ability to cope with oxidative challenges greatly varies between individuals and is associated with individual traits such as age, breeds, behavior, or social rank.

### 2.1. Sperm Metabolism

As other cells, the male gametes produce ROS as a result of their aerobic metabolism (Table 1). In a healthy cell, it is estimated that approximately 2% of O_2_ is converted to ROS. Spermatozoa, precisely bovine, were actually the first cells to be recognized as capable of generating and eliminating ROS [29]. This crucial finding was then confirmed in spermatozoa from other species such as boar, ram (*Ovis aries*), stallion, and rabbit (*Oryctolagus cuniculus*) [30,31]. Within the animal cell, the mitochondrion is the main ROS-producing organelle: complexes I and III are a major source of ROS, while minor sources include lipoic acid-containing oxidative decarboxylation reactions, pyruvate, and 2-oxoglutarate dehydrogenases, among others [32,33]. In addition, sperm membrane-associated reduced nicotinamide adenine dinucleotide phosphate (NADPH) oxidase (in stallions: [34]) and aromatic amino acid oxidase (in bulls: [35]; in rams: [36]; in stallions: [37]) have shown to generate ROS in domestic animals. Among the ROS involved in sperm function, ^•^NO occupies a prominent role as long as it is not only a free radical but also a gasotransmitter [38]. The ^•^NO is synthesized from L-arginine by three isoforms of NO synthase (NOS), whose expression and localization in the sperm cell vary among mammalian species [38]. In addition to ROS produced by sperm cells, it is important to note that interactions among ROS can generate new ROS: for instance, peroxynitrite (ONOO^−^) is generated when ^•^NO reacts with O_2_^•−^and has been shown to induce oxidative stress in porcine spermatozoa [39].

### 2.2. Leukocytes and Immature, Abnormal, or Dead Spermatozoa

Other important sources of ROS within the ejaculate are leukocytes, particularly neutrophils, as well as immature, abnormal, and dead spermatozoa [31,40,41]. Considering that teratospermia (over >60% abnormal spermatozoa in the ejaculate) commonly occurs in the majority of felid species or subspecies [42], abnormal sperm cells might represent a relevant source of oxidative stress in this taxonomic group. In other species, such as horses, it has been shown that abnormal spermatozoa generate higher levels of ROS (i.e., H_2_O_2_) than normal spermatozoa [31]. Surprisingly, Gibb et al. [43] more recently found that the most fertile stallions are paradoxically those having ejaculates with increased oxidative DNA damage and lower sperm concentration, viability, and acrosome integrity. This phenomenon might be explained by the fact that the metabolism of stallion spermatozoa almost entirely relies on oxidative phosphorylation for the generation of ATP for motility in contrast to the more glycolytic metabolism of human or rodent spermatozoa; therefore, oxidative stress might be the result of the intense mitochondrial activity of equine spermatozoa [43]. Additionally, in boars, a high percentage of dead spermatozoa in raw semen significantly increases ROS generation and DNA fragmentation in frozen–thawed samples [41]. In bulls [35], rams [36], and stallions [37], dead sperm cells show enhanced activity of aromatic amino acid oxidase, which in the presence of the amino acid substrate, H_2_O, and O_2_ generates H_2_O_2,_ ammonia (NH_3_), and the corresponding keto acid. Although neutrophils are not commonly observed in sperm samples from livestock species [31,40], their concentration might increase following infections or inflammations of the genital tract, as well as after centrifugation for the removal of seminal plasma or blood contamination during epididymal sperm recovery. In addition to ROS associated with leukocyte activation, bacteria themselves can generate oxidative stress, thereby representing an extrinsic source of ROS in the male reproductive system [44]. Remarkably, few studies (in rabbits: [45,46]; in pigs: [47]) have investigated the role of bacterial contamination in the sperm oxidative status in animals other than humans or laboratory rodents.

Among sperm defects, the presence of cytoplasmic droplets has been indicated as a major intrinsic source of ROS for the sperm cells [48], although other authors suggest that it may protect against oxidative stress due to the antioxidants found in the residual cytoplasm [49]. Whether indeed ROS levels are significantly associated with the proportion of sperm cells with cytoplasmic droplets in humans [48], empirical studies in other mammalian species do not find unequivocal support [49,50]. For instance, the levels of lipid peroxidation are positively associated with the incidence of proximal cytoplasmic droplets but negatively to that of the distal cytoplasmic droplets in bovine spermatozoa [49]. In boars, Matás et al. [50] found that ROS generation is lower in epididymal than in ejaculated spermatozoa, although the incidence of cytoplasmic droplets is commonly higher in epididymal samples than in the ejaculates. The role of such residual cytoplasm against oxidative stress is especially relevant when samples are directly collected from the epididymal caudae since the presence of a distal cytoplasmic droplet is a normal morphological feature of the sperm cells at this stage of maturation. Sperm collection from epididymides represents a very useful source of gametes from highly genetically valuable domestic or wild animals that undergo castration or unexpectedly die. Moreover, storage conditions may influence the incidence of cytoplasmic droplets in epididymal spermatozoa: epididymides stored at 4 °C show a lower incidence of distal cytoplasmic droplets and improved in vitro fertilization (IVF) outcomes compared to those stored at 34 °C, although the levels of lipid peroxidation did not vary between treatments [49]. In dogs, the incidence of sperm cells without cytoplasmic droplets was significantly higher in the epididymal cauda than in the caput, but there were no differences in enzymatic antioxidant activities among epididymal regions ([51]; see also [52]).

### 2.3. Individual Traits

Other factors that may affect ROS production are related to individual characteristics such as age, genetic traits, social factors, or fertility status. Oxidative stress is a known factor associated with aging that may contribute to explain the reduced reproductive performance (e.g., low sperm concentration and motility) observed in senior individuals (in bulls: [53]; in stallions: [54]). The reduced sperm quality associated with aging is accompanied by changes in the lipid profile (e.g., decreased PUFA content), reduced antioxidant defense, changes in mitochondrial function, and increased ROS production [54,55]. In bulls, for instance, aging is associated with reduced post-thaw sperm quality (lower sperm motility and mitochondrial activity) and increased oxidative damage to lipids and DNA [53]. In contrast to these findings, Vince et al. [56] found that seminal plasma of young bulls shows higher protein carbonyl content and lower total sperm number per ejaculate compared to those of old individuals, although the latter show lower activities of antioxidant enzymes, such as total superoxide dismutase (SOD), copper/zinc superoxide dismutase (Cu/Zn SOD), and total glutathione peroxidase (GPx). Age-related decline of sperm quality associated with increased oxidative damage has also been observed in the red junglefowl (*Gallus gallus*), where old males showed lower levels of seminal antioxidants and increased sperm DNA damage than young ones [57]. In contrast to these findings, ROS levels do not differ across ages and fertility status in the canine semen [58], although Domoslawska et al. [59] found lower total antioxidant capacity and higher protein oxidative damage in infertile than in fertile dogs. In horses, which similarly to dogs, have a long breeding lifespan and are typically not selected for their fertility. Unlike other livestock species, sub-fertile males show lower levels of sperm oxidized proteins during the breeding season and lower levels of oxidized lipids during the non-breeding season, compared to the fertile individuals [60]. Moreover, a great individual variability has been observed in the ability of stallion sperm cells to produce ROS, such as ^•^NO and O_2_^•−^, which also vary depending on external factors, such as season or handling conditions [61,62]. This might, at least partly, explain the large inter-individual variability in the levels of lipid peroxidation observed after sperm cryopreservation in this species [63]. In addition, ROS levels may also vary within ejaculate fractions: for instance, Li et al. [64] found that frozen–thawed boar spermatozoa collected from the post-sperm rich fraction (post-SRF) generate more O_2_^•−^ and H_2_O_2_ than those obtained from the SRF of the ejaculate. Unlike livestock and pets, the implications of oxidative stress on the sperm quality of non-domestic animals are largely unexplored. In the seminal plasma of the Asian elephants (*Elephas maximus*), Satitmanwiwat et al. [65] found that high levels of lipid peroxidation and protein carbonyl content were associated with poor sperm motility. These findings may be very useful for the optimization of assisted reproductive techniques (ARTs), which can contribute to the conservation of endangered species.

Interestingly, the male ability to cope with oxidative stress is associated with the development of secondary sexual characters such as plumage traits in birds (carotenoid-based ornaments in the great tits, *Parus major*: [66]; melanin-based ornaments in house sparrow *Passer domesticus*: [67]) or wing spot characteristics in insects (*Hetaerina americana*: [68]). On the other hand, Tomášek et al. [69] found that the intensity of beak coloration (a carotenoid-based ornament) predicted an increase in sperm velocity under control conditions and a decline in sperm velocity under oxidative challenge in the zebra finch (*T. guttata*). Additionally, the social rank may influence the ability of the male to withstand oxidative stress. In house sparrows (*P. domesticus*), it has been found that subordinate males produce less-oxidized and higher-quality ejaculates than dominant males; however, under oxidative stress, they unexpectedly suffered the largest decline in sperm velocity [70,71].

## 3. Extrinsic Factors Associated with Oxidative Stress in the Male Reproductive System

A wide variety of extrinsic factors, the majority of which have a recognized anthropogenic origin, can induce oxidative stress in the male reproductive system [72] (Table 1). Climate change, pollution, urbanization, reduced habitat quality, emerging diseases, and direct human disturbance are factors that may disrupt, simultaneously and potentially synergize with, the oxidative status of animals with negative consequences on their fitness and reproductive success [73].

### 3.1. Climate Change

It is becoming increasingly evident that global warming represents a current and growing threat to animal biodiversity and a major economic challenge for the livestock breeding systems [74,75,76]. Both in vertebrates and invertebrates, climate change may alter the animal oxidative status and fertility rates directly by heat stress or indirectly by provoking water and food shortage, ocean acidification, or increasing the toxicity of pollutants [75]. In most mammalian species, spermatogenesis requires an optimal temperature that is usually 2–5 °C below the core body temperature. The maintenance of testicular temperature within optimal values is guaranteed by a complex system of anatomical structures that include the dartos, the pampiniform plexus, the cremaster muscle, and sweat glands, which are distributed over the scrotal skin. Whenever the testicular temperature exceeds the scrotal thermoregulatory capacity, heat insult occurs and negatively affects male fertility [77]. Among domestic animals, cattle and pigs are probably the most sensitive species to heat stress or at least those in which the phenomenon has been more extensively studied. In bulls, a short period of heat stress (two days) can lead to a prolonged period (one month or more) of reduced sperm quality, with the male germ cells at meiotic and post-meiotic stages of development being the most susceptible to high temperatures [78].

### 3.2. Seasonality

In species in which spermatogenesis occurs all year round, such as boars and bulls, seasonality represents another extrinsic factor that may affect male fertility by increasing ROS levels and/or reducing the antioxidant defenses. In this context, Vince et al. [56] found that oxidative stress markers and antioxidant defenses of bull spermatozoa differ depending on the time of the year being, for example, the protein carbonyl content higher and selenium-dependent glutathione peroxidase (Se-GPx) activity lower during the warm season. However, variations in ROS levels in the bull semen throughout the seasons might be affected by the geographic location [79]. In Sweden, for instance, Valeanu et al. [80] only found a slight decline in bull sperm quality and an increase in ROS levels during the summer. In stallions, the sperm motility is higher, and the ROS levels are lower at the end than at the beginning of the breeding season [62]. More recently, Mislei et al. [81] found that stallion semen shows higher lipid peroxidation levels and H_2_O_2_ production in winter than in summer, while mitochondrial O_2_^•−^ production was higher in summer than in winter.

### 3.3. Radiation

The exposure of sperm cells to a light source has been shown to increase ROS levels, the amount of which varies depending on the wavelength employed [82]. Typically, light at short wavelengths increases ROS generation, while at long wavelengths it has beneficial effects on sperm function, fertilizing ability, and resilience to the preservation process [83]. In this way, ultraviolet (UV) and blue light irradiation are powerful sources of ROS, both in ram and tilapia (*Oreochromis aureus*) spermatozoa [82]. In stallion spermatozoa, Ghosh et al. [84] recently found that UV light induces lipid peroxidation irrespectively of the media and assays used. Additionally, the exposure to ionizing radiation, which arose after the nuclear power plant accidents of Chernobyl (Ukraine) in 1986 and Fukushima Daiichi (Japan) in 2011, has provoked a decline in richness and abundance of several animal species that may at least partly be mediated by the impact of oxidative stress on reproductive function [85,86]. To date, however, no negative effects on spermatogenic and sperm function have been observed in wildlife (in raccoons, *Procyon lotor*: [87]) and livestock (bull: [88]) inhabiting the contaminated areas closed to Fukushima, probably as a result of different exposure features and reduced multigenerational mutation accumulation in the latter compared to Chernobyl’s fauna [85,89]. In contrast, the effect of exposure to non-ionizing and non-visible radiation (e.g., radio waves and microwaves) in sperm cells from domestic and wild animals is largely unknown, although the chances of exposure to this type of electromagnetic waves have been increasing over the last decades as it occurs, for instance, during the routine security checks required for air transportation of biological material such as artificial insemination doses [90]. In this study, Tirpak et al. [90] found that the exposure to radio waves (93 kHz) produced by metal detectors had some negative effect on sperm motility, but it remains to be investigated if they were the result of oxidative damage. In a similar way, light pollution associated with urban areas has been suggested to impair the blood oxidative status of wildlife [91], although its implications on male reproduction are still unexplored.

### 3.4. Chemical Pollutants

An increasing number of studies indicate that oxidative stress is an important mechanism of heavy metals’ toxicity for the male reproductive system both in vertebrates [92] and invertebrates [93]. Heavy metals such as lead (Pb), cadmium (Cd), and mercury (Hg) stimulate ROS overproduction, which disrupts the antioxidant defenses and provokes oxidative damage to lipids, proteins, and DNA, which eventually culminate in cell apoptosis or necrosis [94]. Among heavy metals, Pb is viewed as a persistent global threat based on the millions of tons of Pb mined in the past and recent years [95]. Sources of Pb exposure for wildlife are related not only to mining activities but also to urbanization (exhaust of vehicles), hunting (ammunitions), and fishing (fishing weights) activities [95]. Moreover, the exposure to endocrine-disrupting chemicals has been shown to have a negative impact on male fertility that is, at least partly, mediated by oxidative stress in humans and laboratory animals [96], despite limited knowledge being available for other animal species [97]. In the spirlin (*Alburnoides bipunctatus*), Kocabaş et al. [98] have recently found that bisphenol A induces a dose-dependent decrease in sperm motility, SOD, and GPx activities, which were associated with increased levels of lipid peroxidation and catalase (CAT) activity. In a recent study, Tartu et al. [99] found that in blue whale (*Balaenoptera musculus*) and fin whale (*Balaenoptera physalus*), persistent organic pollutants such as polychlorinated biphenyls (PCBs) or organochlorine pesticides (OCPs) show higher levels in males than in females, but little is still known about their role and consequences on male reproductive function. Other pollutants such as per- and polyfluoroalkyl substances (PFAs), which are used in the industrial production of several products (e.g., pans, clothes, cosmetics) and are highly persistent in the environment, have shown a negative effect on male reproduction [96]. In male lizards (*Eremias argus*), the exposure to PFAs, as it occurs in perfluorooctanoic acid-contaminated soil, decreases testes mass, while it increases testicular enzymatic antioxidants (i.e., SOD and CAT) and oxidative stress markers [100]. In boars, perfluorooctane sulfonate and perfluorohexane sulfonate, which are other common PFAs, have been shown to induce ROS overproduction, increase DNA fragmentation, and alter the patterns of protein tyrosine phosphorylation during sperm capacitation [101].

### 3.5. Human Disturbance

In recent years, human disturbance associated with the eco-tourism has also shown a negative impact on wildlife abundance and breeding success (e.g., in the bottlenose dolphins *Tursiops* sp.: [102]; in the yellow-eyed penguins *Megadyptes antipodes*: [103]; in the California sea lions *Zalophus californianus*: [104]). Although the implications of these human activities on male reproduction in wildlife are still minorly explored, eco-tourism has been shown to affect the male behavior in the Barbary macaques (*Macaca sylvanus*; [105]), while in the male marine iguanas (*Amblyrhyncus cristatus*), it increases body mass, reactive oxygen metabolites, and baseline corticosterone and testosterone levels [106].

### 3.6. Iatrogenic Damage Associated with ARTs

Other extrinsic sources of ROS might be related to the iatrogenic damage caused by sperm handling and storage: oxidative stress may be generated during each step of in vitro manipulation for ARTs, including exposure of spermatozoa to thermal changes, centrifugation, and freezing–thawing procedures [107,108] (Table 2). Cutting-edge ARTs such as flow cytometric sperm sorting are able to induce [109] or increase [22] the susceptibility of sperm cells to oxidative stress. However, different results have been reported across species, or even within the same species, regarding the impact of ARTs on the sperm redox status. For instance, the freezing–thawing process has been shown to induce oxidative stress in sperm cells from stallions [31,61,81,110], bulls [111,112,113,114], and cats (*Felis catus*) [115]; however, there is still controversy whether this ART induces oxidative stress in sperm cells from pigs [14,16,115,116,117,118,119] and dogs [58,120,121]. In buffalos, Kadirvel et al. [122] and Lone et al. [123] found that cryopreservation increases sperm lipid peroxidation, whereas conflicting results were observed on ROS levels. Additionally, in domestic birds, sperm cryopreservation induces oxidative damage by increasing the lipid peroxidation levels [124]. In turkeys (*Meleagris gallopavo*), Słowińska et al. [125] found that ROS levels significantly increased after freezing–thawing and after 24 h of semen liquid storage (4–7 °C). Different results have also been reported at the intra-specific level: in boar sperm cells, for instance, Awda et al. [14] found that cryopreservation decreases intracellular O_2_^•−^, while it does not affect H_2_O_2_ levels in the viable sperm cells; in contrast, Kim et al. [116] and Yeste et al. [117] found the that the freezing–thawing process increases the percentage of sperm cells with high H_2_O_2_ levels, while it does not affect that of high intracellular O_2_^•−^ levels. This lack of consensus between studies may be linked to the differences in experimental design, laboratory assays, or the methods used to indicate the oxidative stress as, for instance, the percentage of sperm cells with high ROS levels or the mean fluorescence intensity per total or viable cells. Moreover, ideally, both ROS levels and antioxidant defenses should be considered in order to determine whether an insult can induce oxidative stress. In another study, Yeste et al. [118] found that the H_2_O_2_ levels in viable boar sperm cells increase after freezing–thawing, irrespective of the ejaculate’s cryotolerance, although the increased values were detected only after 30 min of post-thawing incubation and not thereafter. Similarly, Gómez-Fernández et al. [126] found that seminal post-thaw ROS levels and lipid peroxidation are similar between boars regarded as “good freezers” or “bad freezers”. In contrast, Llavanera et al. [127] more recently found that post-thaw intracellular O_2_^•−^ levels in boar sperm cells were higher in “poor freezers” than in “good freezers”. Intra-specific differences might be related to several factors, including a male-dependent effect: for instance, stallion sperm cells overall increase ^•^NO levels after freezing–thawing, although in a few males, none or even the opposite trend was observed [61]. Within species, the type of ROS produced may also vary depending on the conditions of cell storage (e.g., in bulls: [111]; in boars: [116]). In bovine, sperm cooling and freezing–thawing increase the levels of O_2_^•−^, while they do not affect the levels of H_2_O_2_; by contrast, ^•^NO does not change during cooling but considerably increases after thawing [111]. However, in the same species, Gürler et al. [113] more recently found that all ROS parameters increase after freezing–thawing, although the rise during post-thawing incubation was more remarkable for H_2_O_2_ levels, which may be related to the reduced activity of GPx. Additionally, liquid storage of semen at 4–5 °C has been reported to increase ROS and lipid peroxidation levels in horses [128], buffalos [122], pigs [129], sheep [130], and goats (*Capra hircus*) [131]. In cats, sperm samples collected from epididymides stored at 5 °C for up to 72 h do not show any change in the levels of oxidative damage to lipids and proteins [132]. The increased ROS levels induced by sperm storage might also be affected by the extender composition [110,111], its oxygenation levels [133], as well as by the presence [61] or type of cryoprotectant [134] used. In bull spermatozoa, Taşdemir et al. [134] found that the type of cryoprotectant affects the sperm antioxidant defenses with, for instance, GSH content and GPx activity being higher in samples cryopreserved with 6% dimethyl sulfoxide (DMSO) than with 6% glycerol, despite the latter providing the greatest sperm motility and DNA integrity after thawing. In an interesting study, Burnaugh et al. [135] found that the osmolality of media also stimulates ROS generation in the sperm cells: the exposure of equine spermatozoa to anisosmotic conditions increases O_2_^•−^ generation and alters protein tyrosine phosphorylation. In dogs, the addition of glycerol during sperm cryopreservation has been shown to induce oxidative stress by increasing sperm lipid peroxidation levels [121]. Additionally, in rams [136] and alpacas [137], ROS levels were significantly higher during the cooling step at 5 °C than after thawing, probably as a result of the cryoprotectant supplementation at this stage (i.e., glycerol and dimethylacetamide, respectively). Iatrogenic oxidative stress can also be induced by sperm selection procedures: for instance, density gradient and centrifugation protocols may increase ROS generation [50,138] and induce oxidative damage to the sperm cells [139]. There is also some controversy about whether sperm selection procedures may select the sperm fraction with reduced ROS levels. In this sense, it has been found that the intracellular ROS generation does not change after gradient centrifugation in frozen–thawed boar spermatozoa [41], whereas single layer centrifugation (SLC) selects motile spermatozoa that contain less O_2_^•−^ and H_2_O_2_ than uncentrifuged controls in stallions [62]. In goats, SLC performed after freezing–thawing improved sperm quality but also increased O_2_^•−^ levels compared to those of spermatozoa selected before freezing: this phenomenon might be explained by the fact that ROS generation may be the result of a more intense metabolic activity of frozen–thawed spermatozoa [140]. Interestingly, sperm handling may also result in oxidative DNA damage due to the presence of metals (particularly iron Fe, aluminum Al, and copper Cu) in the preparation media, as it has been found in humans [141]. Similarly, H_2_O_2_ generation may arise as a result of the oxidative deamination of certain amino acids contained in the egg yolk [30], which is a common additive used for sperm preservation. More recently, Aitken et al. [37] found that following the damage caused by cryopreservation on the sperm plasma membrane, intracellular aromatic amino acid oxidase becomes readily available to interact with aromatic amino acids found in the egg yolk-based media, which finally result in increased H_2_O_2_ levels.

### 3.7. Bacteriospermia

As previously mentioned, bacteria can indirectly induce oxidative stress by stimulating immunologic defenses such as leukocyte activation and fever [45]. Additionally, bacteria can directly induce ROS overproduction through their toxins and metabolites or by attaching to the sperm cells and activating the signaling pathways related to oxidative stress [44]. However, our knowledge about the implications of the last mechanism in the reproductive performance of domestic and wild animals is still in its infancy. In domestic and wild animals, the presence of bacteria in the semen may arise following infections of the genital tract or, more frequently, during sperm collection and handling. The bacterial contamination induced by in vitro inoculations of microorganisms (e.g., *Enterococcus faecalis*) or their products (outer membrane vesicles of *Proteus mirabilis*) have been shown to induce ROS overproduction and decrease sperm quality [46,47]. Interestingly, in porcine sperm, Gao et al. [47] found that inoculation of outer membrane vesicles of *Proteus mirabilis* increased the sperm mitochondrial membrane potential, which was associated with increased expression of apoptosis- and autophagy-related proteins.

## 4. Antioxidant Defenses of the Male Reproductive System

The primary enzymatic ROS scavengers described in semen are CAT, SOD, GPx, glutathione reductase (GR), glutathione S-transferase (GST), and peroxiredoxins [144]. While the body owes enzymatic defenses against O_2_^•−^ (i.e., SOD) and H_2_O_2_ (i.e., CAT, GPx, and peroxiredoxins), there is no known enzymatic system that eliminates ^•^OH, probably because of its high reactivity [3]. A number of other non-enzymatic compounds such as GSH, albumin, urate, taurine, hypotaurine, pyruvate, lactate, ascorbic acid, tocopherol, and L-ergothioneine are present in seminal plasma and may act as antioxidants and counteract oxidative stress [144]. In horses, other seminal components such as the seminal plasma proteins 1 and 2 (HSP-1/2) have been shown to inhibit lipid peroxidation and protect enzymes such as alcohol dehydrogenase and glucose-6-phosphate dehydrogenase by acting as a chaperone against oxidative stress [145]. On the other hand, Leahy et al. [22] found that the addition of seminal plasma proteins in the pre-freezing media better preserves sperm quality after freezing–thawing, although it may also increase ROS generation in ram spermatozoa.

Numerous factors such as genetics, physiological status, age, management, and climatic conditions may influence the antioxidant status of the ejaculate, most of which have not been fully elucidated yet [56]. In bulls, for instance, the antioxidant defenses of the ejaculate vary depending on factors such as the type of antioxidant, age of the male, or time of the year [56]. Additionally, in poultry, sperm antioxidant defenses and lipid peroxidation levels significantly vary among and within breeds [146]. There are also considerable differences across species in the amount of seminal antioxidant defenses, for instance, the CAT content being very abundant in rabbits (on average 275 µg/mL) and extremely scarce in boars (on average 0.2 µg/mL) [147]. This helps to explain why the ability of sperm to eliminate a type of ROS differs greatly across species: boar spermatozoa are indeed the least efficient to eliminate H_2_O_2_, whereas rabbit and stallion spermatozoa are the most efficient ones [30]. In addition, in avian species, the enzymatic antioxidant defenses vary between species, for example, SOD activity being greater in goose than in chicken spermatozoa [148]. Within species such as pigs, the antioxidant capacity of the seminal plasma, which is associated with enhanced sperm quality [149] and fertility [150], not only varies between and within males, but also between ejaculates and ejaculate’s fractions [64,150]. The scavenging potential of the seminal plasma depends on a variety of antioxidants that originate from various fluids of the male reproductive tract. In boars, for instance, CAT activity in fluids from the prostatic gland is two- to threefold higher than those from the epididymal tails or vesicular glands [151]. In the same species, Park et al. [152] found that SOD1, GPx, and peroxiredoxin 5 (PRDX5) levels change during sperm maturation, increasing as the spermatozoa move from the caput to the cauda of epididymis. Additionally, in goats [153] and European bison (*Bison bonasus*) [154], SOD activity has been found to be higher in the epididymal cauda than in the corpus and caput. In contrast, SOD and GPx activities do not differ among epididymal regions in dogs [52]. Antioxidant defenses may also vary within ejaculate fractions: for instance, the high sperm quality of the boar SRF is associated with higher SOD and paraoxonase-1 (PON-1) activities than those of the post-SRF and recomposed ejaculate [64]. Similarly, the greatest values of SOD activity were found in the SRF of dog ejaculate, whereas low or undetectable values of CAT activity were observed in each ejaculate fraction [52,155].

Factors related to sperm handling, such as extender type, storage conditions, or sample homogenization may also affect the efficiency of antioxidant defenses [129,131,142,143] (Table 2). In stallion ejaculates, for instance, the activity of antioxidant enzymes is affected by the semen dilution but not by liquid storage at 5 °C [143]. In this study, Kankofer et al. found that the activity of GPx, SOD, and CAT, as well as malondialdehyde (MDA) levels, were higher in extended semen than in raw semen, which might be related to the interaction between the extender and the seminal plasma components [143]. In boars, SOD and GPx activities change during storage in non-homogenized semen, being significantly higher at 168 h than 72 h of storage at 17 °C [129]. On the other hand, enzymatic activities do not change during storage time in samples that were homogenized twice a day [129]. In caprine semen, Liu et al. [131] recently found that the activity of mitochondrial GPx, SOD, and CAT significantly decrease during 96 h of liquid storage at 4 °C. For this purpose, the activity of antioxidant enzymes can also be used as cryotolerance sperm markers by protecting sperm cells from oxidative stress induced by cryopreservation. For instance, SOD activity is higher in the donkey (*Equus asinus*) and stallion seminal plasma of “good freezability” than in “bad freezability” ejaculates, while CAT, GPx, and GR activities do not differ between groups [156,157]. Although the knowledge about potential antioxidant markers of sperm cryotolerance in wildlife is limited, high radical reduction capacity of the seminal plasma is associated with greater sperm motility and viability after freezing–thawing in African lions (*Panthera leo*) [158].

## 5. Effects on Oxidative Stress on Male Reproduction in Domestic Animals

**Figure 1:**
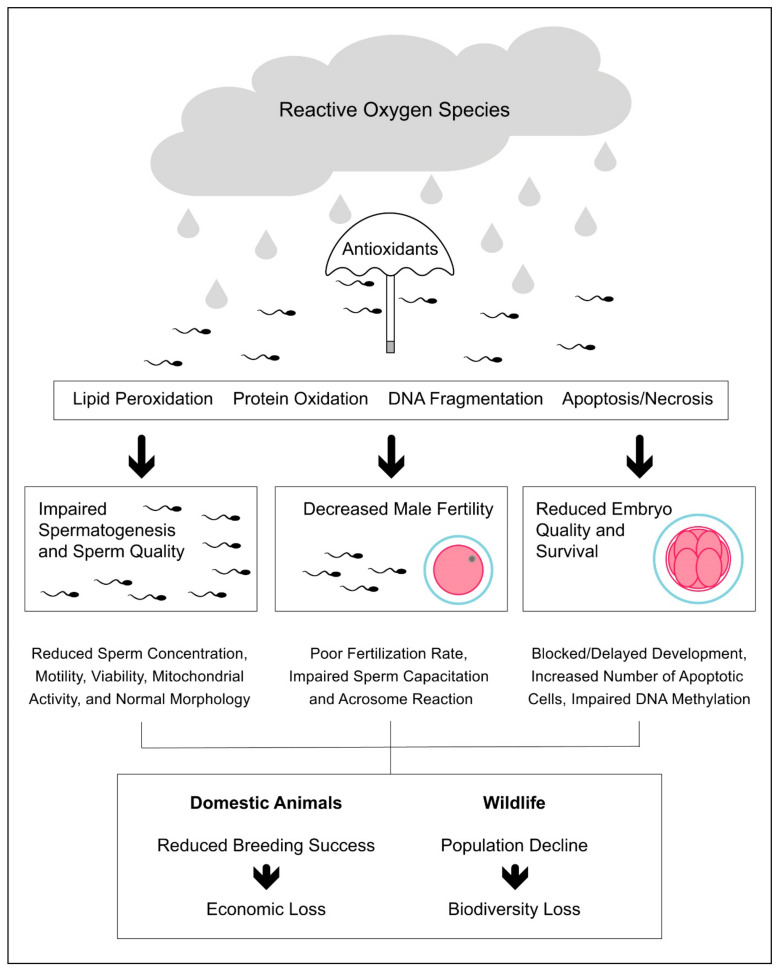
Impact of oxidative stress on male reproductive performance in domestic and wild animals.

In the livestock industry, the impact of oxidative stress on male reproduction is not only associated with reduced sperm quality and fertility [82,159] but also with reduced offspring health, especially in terms of embryo quality and survival [160,161] (Figure 1). In cattle, Simões et al. [161] found that the susceptibility of spermatozoa to oxidative stress is associated with decreased sperm DNA integrity and embryo cleavage rate. The authors also found that the increased susceptibility to oxidative stress did not affect the number of blastomeres, while it significantly increased the number of embryonic apoptotic cells [161]. More recently, Wyck et al. [162] found that bull spermatozoa exposed to oxidative stress induced by H_2_O_2_ showed increased DNA damage, which was associated with impaired DNA demethylation during epigenetic reprogramming in early embryonic development. In a recent systematic review, Rybas-Maynou et al. [163] found that the impact of oxidative stress at the sperm level translates into poorer fertilization and blastocyst rates after ARTs, such as IVF or intracytoplasmic sperm injection (ICSI).

The oxidative stress induced by exposure to elevated environmental temperatures has shown a negative impact on livestock reproduction and is responsible for the decline in reproductive performance observed during heat waves in both sexes [164]. To date, the consequence of heat stress on the male fertility in livestock has been mainly explored in bulls [77], in which it provokes a decrease in sperm motility and viability and an increase in sperm abnormalities [78]. In the same study, the authors also found differences between breeds regarding the negative impact of heat stress on sperm morphology being, for instance, the effects more pronounced in Belgian blue than in Holstein-Friesian bulls. Such a difference might be related to the lower scrotal thermoregulatory capacity of the Belgian blue bulls due to their extreme muscularity combined with the small scrotal size [78]. In a recent study, Garcia-Oliveros et al. [165] found that the impact of heat stress on bull sperm quality changes throughout the time following the heat-injury: for instance, it first increases sperm morphological abnormalities (7 days following heat stress), then lipid peroxidation (14 days following heat stress), and finally DNA fragmentation (28 days following heat stress). In contrast, Llamas-Luceño et al. [166] found that heat stress decreases sperm viability, while it does not affect other parameters such as motility, ROS levels, DNA fragmentation, and lipid peroxidation. Interestingly, the authors also found that heat stress had a negative impact on blastocyst development (i.e., decreased blastocyst rate and delayed hatching), which may generate important economic loss for companies dedicated to embryo production and transfer [166].

The presence of pollutants, such as pesticides [167] or heavy metals [168], has been detected in the semen of several domestic animal species. In bovine semen, Pb and Cd levels from environmental origin are positively related to ROS levels and lipid peroxidation and are negatively associated with sperm motility, CAT activity, and GSH levels [92]. Short exposure to Hg decreases bull sperm quality (motility, membrane, and DNA integrity) and fertilizing capacity by increasing ROS and lipid peroxidation levels [169,170]. In the same species, it was also found that Hg impairs antioxidant defenses by decreasing sperm total antioxidant capacity and GSH levels [169,170]. Similarly, in boars, persistent organic pollutants, such as perfluorooctane sulfonate and perfluorohexane sulfonate, induce oxidative stress and impair sperm capacitation by decreasing tyrosine phosphorylation in proteins of the sperm head equatorial and acrosomal regions [101].

Liquid and frozen storage of sperm cells induce damages to proteins, lipids, and nucleic acids that are at least partly mediated by oxidative stress [144]. In a recent study in goat spermatozoa, Liu et al. [131] found that oxidative stress caused by prolonged liquid semen storage accelerates mitochondria-dependent apoptosis by activating mitogen-activated protein kinase (MAPK) signaling pathway-related proteins, such as c-Jun N-terminal kinase (JNK) and protein kinase A (PKA), and downregulating the expression of antioxidant enzymes, such as SOD2 and peroxiredoxin 1 (PRDX1). The increased ROS levels induced by the freezing–thawing process are associated with carbonylation of several proteins that are involved in sperm capacitation [114], which might be related to the capacitation-like changes and increased value of acrosome-reacted spermatozoa observed in cryopreserved samples [21]. Besides, in some species, the ability of sperm cells to produce ROS is associated with their capacity to withstand the freezing–thawing process (in stallions: [171]; in boars: [127]; see also [118,126]). Interestingly, Yeste et al. [171] found that the intracellular sperm ROS levels are higher in the stallions with good-freezability ejaculates than those with poor-freezability ejaculates, although they do not differ in the sperm DNA integrity. In boars, Llavanera et al. [127] found that spermatozoa with high post-thawing O_2_^•−^ levels are associated with high pre- and post-cryopreservation levels of glutathione S-transferase Mu 3 (GSTM3), a membrane-bound isoenzyme whose expression levels are negatively associated with porcine litter size [172]. In addition, sperm selection procedures, such as centrifugation, have been shown to impair stallion sperm motility and mitochondrial functionality and to induce oxidative damage to DNA and proteins, the effect being dependent on the time and centrifugal force applied [139]. In the pig industry, the common practice of rotating the artificial insemination doses to prevent cell sedimentation has been proposed as a likely source of oxidative stress, which might be responsible for the decline in sperm quality during prolonged storage at 17 °C [173]. In this context, Menegat et al. [129] have recently shown that twice-a-day homogenization does not affect the oxidative status of boar sperm cells stored at 17 °C for up to 7 days. In the same study, the authors also found that homogenization does not affect SOD activity, although it significantly increases GPx activity at 72 h of liquid storage compared to non-homogenized samples.

## 6. Effects of Oxidative Stress on Male Reproduction in Wildlife

Oxidative stress is the unifying feature underlying the toxicity of anthropogenic pollution [174], which has shown a negative effect on wildlife reproduction [175]. Like domestic animals, the oxidative stress induced by anthropogenic factors can decrease the male reproductive performance and success, thus contributing to wildlife population decline (Figure 1). Pollutants originating from human activities have reached the most remote places on Earth, from the bottom of Mariana Trench [176] to the top of Mount Everest [177]. Using a meta-analytical approach, Isaksson [174] found that animals living in a polluted environment have higher oxidative stress than animals living in a less-polluted environment, the oxidative response to pollution being stronger in birds than in mammals, amphibians, or insects. In one of the most polluted areas of Northern Africa and the Mediterranean Sea, Amri et al. [178] found that hybrid sparrows (*P. domesticus × P. hispaniolensis*) show testicular histopathological lesions, increased levels of lipid peroxidation, and reduced antioxidant defenses compared to birds from control sites.

In red deer living in polluted-mining areas, exposure to Pb negatively affects male reproduction by increasing sperm DNA fragmentation and decreasing sperm motility, viability, and acrosome integrity [179,180]. Mining pollution has been shown to impair SOD and GPx activities in both red deer testes and spermatozoa, which might be a consequence of Pb implications in the Cu and Se homeostasis [179,180]. In addition, testicular levels of non-enzymatic antioxidants such as vitamins A and E were reduced in red deer inhabiting Pb polluted areas [181]. Interestingly, red deer from mining-polluted areas show higher testes mass than those from the control areas, which may suggest a compensatory mechanism whereby spermatogenic activity and sperm production increase to cope with the decreased sperm quality [179,180]. In blue tits (*Cyanistes caeruleus*), exposure to heavy metals reduces the number of spermatozoa found in the perivitelline layer, which may indirectly suggest the negative effect of environmental pollution on male fertility [182]. More recently, Vallverdú-Coll et al. [183] found that sub-lethal oral Pb administration decreases acrosome integrity and impairs sperm motility, while it did not affect sperm viability and fecundation rate in the red-legged partridges (*Alectoris rufa*). The negative effects of Pb on sperm quality seem to be modulated by antioxidant levels, as they were observed only when antioxidants were considered as covariates in the statistical analysis [183]. In the testes of freshwater crab (*Sinopotamon henanense*), Wang et al. [93] found that Cd exposure induces oxidative stress and increases lipid peroxidation and the number of apoptotic germ cells. The apoptosis of germ cells might be mediated via the mitochondrion-dependent pathway through regulating caspase-3 and caspase-9 activities [93]. In another invertebrate, the earthworm (*Eisenia fetida*), exposure to petroleum-contaminated soil has been shown to decline reproduction by increasing DNA damage in the seminal vesicles and decreasing enzymatic antioxidant activities of CAT, SOD, and peroxidase (POD) [184].

As with humans, wildlife reproduction also suffers from the negative consequences of ionizing radiation. The consequences of this type of radiation on male reproduction of wild animals have been mainly studied after the power plant accidents that occurred in Chernobyl and Fukushima. In birds breeding in Chernobyl areas, males show an increased incidence of sperm abnormalities and impaired motility [185,186]. For instance, the incidence of aspermia is around 18% in birds breeding in Chernobyl compared to 3% found in control sites [186]. Interestingly, in the male barn swallow (*Hirundo rustica*), sperm length is shorter in individuals breeding in the radioactively contaminated area compared to the control site [185], the radiation levels being associated with oxidative stress and impaired sperm swimming pattern (i.e., high curvilinear velocity and lateral head displacement) in this species [86,187].

## 7. Strategies for Palliating the Negative Effects of Oxidative Stress on Male Reproduction

Considering the variety of intrinsic factors that are associated with male susceptibility to oxidative stress, an initial first strategy to improve the reproductive management of livestock consists of the selection of genetic characteristics that confer a greater resistance of sperm cells to an imbalance between ROS levels and antioxidant defenses. It is known that most of the livestock production worldwide relies on a short list of breeds, such as the Holstein cattle or the Large White pig, which have been mostly selected for their productive traits. Their weakness, however, relies on their genetic homogeneity, which makes them extremely sensitive to changes in environmental conditions such as heat stress. In this context, global warming induced by climate change may lead to the “re-discovery” of indigenous and more resilient breeds of domestic animals. In wildlife, population decline due to habitat loss or fragmentation contributes to inbreeding that can predispose the males to greater susceptibility to oxidative stress. In this sense, in situ and ex situ conservation programs are essential to preserve wildlife biodiversity and their resilience to environmental changes. The second strategy consists of reducing the levels of oxidative stress in the ejaculate. Several approaches have been proposed for this purpose and will be briefly summarized here. Considering that hundreds of millions of livestock artificial insemination doses are estimated to be sold worldwide yearly, strategies to mitigate the impact of oxidative stress on the sperm quality may have a great economic impact on the livestock industry. The antioxidants supplementation for improving the male reproductive performance of farm animals might be administered through the diet or as an additive to the semen extenders [188]. For instance, the addition of GSH to the semen extender has been shown to improve sperm function and fertilizing ability in mammals [189], birds [190], and fish [191]. The addition of homologous or heterologous seminal plasma has also been proposed as a strategy to improve the antioxidant defense of sperm cells against oxidative stress [192], although different results have been obtained across studies. For instance, in boars, ROS and free thiol levels were significantly higher in thawed semen samples supplemented with 50% seminal plasma volume compared to those supplemented with 10% [193]. More recently, the addition of antioxidants from plant extracts has shown a beneficial effect on porcine sperm quality during liquid storage or under oxidative stress conditions [194,195]. Another interesting approach to reduce oxidative stress was found by Balamurugan et al. [133], who found that partial deoxygenation of extender prior to semen addition decreases post-thaw ROS and lipid peroxidation levels and increases sperm quality in buffalos. Besides O_2_, the regulation of other gaseous molecules might be used to palliate the detrimental consequences of oxidative stress in the semen. For instance, the semen supplementation with ^•^NO inhibitors (i.e., aminoguanidine) and hydrogen sulfide (H_2_S) donors (i.e., GYY4137) have shown beneficial effects in preserving porcine sperm quality under induced-oxidative stress [196,197]. The beneficial effects of antioxidant supplementation prior to semen preservation are useful not only for the management of domestic species (e.g., domestic cat: [198]) but also for the conservation of their wild and endangered relatives (e.g., flat-headed cat, *Prionailurus planiceps*: [199]).

## 8. Conclusions

Like humans, domestic and wild animals are also exposed to a variety of intrinsic and extrinsic sources of oxidative stress that cause reproductive dysfunctions, which may culminate in a compromised fertility and population decline. On the one hand, this can result in a major economic loss for the livestock industry, while on the other hand, it can contribute to biodiversity loss that may further threaten the ecosystem’s health and integrity. Studies investigating the implications of oxidative stress on male fertility in domestic and non-domestic animals as well as the strategies to palliate the deleterious consequences of oxidative damage are needed. The benefits provided by a more comprehensive knowledge in this field are not only limited to the livestock industry but also to biodiversity conservation.

## Figures and Tables

**Table 1:** Known and potential factors associated with oxidative stress in the male reproductive system of domestic and wild animals.

Intrinsic Factors	Extrinsic Factors
*Cellular* Sperm metabolism Mitochondrial enzymes NADPH synthase Aromatic amino acid oxidase ^•^NO synthase Sperm morphology and viability Immature, abnormal, or dead spermatozoa Presence of cytoplasmic droplets Leukocyte activation following inflammation or infection *Individual* Age Genetics and phenotypic traits * Behavior Social rank	*Environmental* Climate change Seasonality Radiation Chemical pollutants * Human disturbance (e.g., eco-tourism) *Iatrogenic* Sperm handling Sperm storage Media composition Sperm selection procedures *Pathological* Bacteriospermia

* Potential sources of oxidative stress in the male reproductive system. NADPH: reduced nicotinamide adenine dinucleotide phosphate; ^•^NO: nitric oxide.

**Table 2:** Impact of assisted reproductive techniques on the sperm redox status in domestic animals.

Procedure	Species	Conditions	ROS Levels *	Antioxidant Defenses	Ref.
Cryopreservation	Pig	60 °C, 5 s washed/unwashed samples	Decreased (O_2_^•−^) or unchanged (H_2_O_2_) in viable spermatozoa	n.e.	[14]
	Pig	37 °C, 30 min Percoll-washed samples	Unchanged	n.e.	[16]
	Pig	37 °C, 30 min, BTS	Increased (H_2_O_2_) or unchanged (O_2_^•−^) in viable spermatozoa compared to diluted semen at 15 °C	n.e.	[116]
	Pig	37 °C, 240 min, BTS	Increased (H_2_O_2_, TD) or unchanged (O_2_^•−^) in viable spermatozoa compared to diluted semen at 17 °C	n.e.	[117]
	Pig	37 °C, 240 min, Duragen and lactose-based extenders	Increased (H_2_O_2_, TD) or unchanged (O_2_^•−^) compared to diluted semen at 17 °C	n.e.	[118]
	Pig	37 °C, 240 min, Modena extender	Increased (H_2_O_2_) compared to diluted semen	n.e.	[119]
	Dog	38 °C, 1 min, AndroPRO™ and CaniPRO™ extenders	Increased or unchanged (O_2_^•−^) depending on male age	n.e.	[58]
	Horse	37 °C, 30 s, INRA 96 extender	Increased (^•^NO) but individual variation	n.e.	[61]
	Horse	37 °C, 24 h, Tyrode’s medium	Increased or unchanged (O_2_^•−^ and H_2_O_2_) depending on the season	n.e.	[81]
	Horse	37 °C, 30 s, 4 media tested	Increased (O_2_^•−^)	n.e.	[110]
	Cattle	38 °C, 5 min, Tris-based medium	Increased (O_2_^•−^ and ^•^NO; TD) or unchanged (H_2_O_2_)	n.e.	[111]
	Cattle	37 °C, 24 h, Tris-based medium	Decreased (O_2_^•−^ and H_2_O_2_) in viable spermatozoa	n.e.	[112]
	Cattle	37 °C, 24 h, Tyrode’s medium	Increased (^•^NO, O_2_^•−^, ONOO^−^, ^•^NO_2_, ^•^OH) or unchanged (H_2_O_2_)	n.e.	[113]
	Cattle	37 °C, 1 min, BIOXcell	Increased (O_2_^•−^)	n.e.	[114]
	Cat	37 °C, 30 s, Tris-based medium	Increased (O_2_^•−^) in viable spermatozoa	n.e.	[115]
	Buffalo	Tris-based medium and Percoll-washed samples	Unchanged (H_2_O_2_) in viable spermatozoa	n.e.	[122]
	Buffalo	Tris-based medium	Increased (H_2_O_2_)	Decreased CAT, GPx, SOD, and TAC	[123]
	Turkey	80 °C, 6 s Ovodyl extender	Increased	n.e.	[125]
	Sheep	38 °C, 45 s Skimmed-milk based extender	Unchanged	n.e.	[136]
	Alpaca	37 °C, 1 min, Skimmed-milk based extender	Unchanged (O_2_^•−^ and H_2_O_2_)	n.e.	[137]
Cooling	Cattle	4 °C, 2 h, Tris-based medium	Increased (O_2_^•−^) or unchanged (^•^NO and H_2_O_2_)	n.e.	[111]
	Cattle	4 °C, 2 h, Tris-based medium	Unchanged (O_2_^•−^) or decreased (H_2_O_2_) in viable spermatozoa	n.e.	[112]
	Pig	5 °C, 1.5 h, BTS and lactose-based extender	Decreased (H_2_O_2_ and O_2_^•−^) in viable spermatozoa	n.e.	[116]
	Pig	4 °C, 3–4 h, Modena extender	Unchanged (H_2_O_2_)	n.e.	[119]
	Sheep	5 °C, 2.5 h, Skimmed-milk-based extender	Increased	n.e.	[136]
	Alpaca	5 °C, 1.5 h, Skimmed-milk-based extender	Increased (O_2_^•−^ and H_2_O_2_)	n.e.	[137]
Cool storage	Buffalo	4 °C, 72 h, Tris-based extender	Increased (H_2_O_2_) in viable spermatozoa	n.e.	[122]
	Turkey	4–7 °C, 48 h Ovodyl extender	Increased	n.e.	[125]
	Pig	17 °C, 168 h, Androstar^®^ Plus and BTS	Increased (H_2_O_2_)	Increased or unchanged (TAC, GPx and SOD)	[129]
	Pig	16 °C, 5 days, 4 media tested	n.e.	Unchanged (SOD)	[142]
	Sheep	4 °C, 96 h, OVIXcell	Increased (H_2_O_2_)	n.e.	[130]
	Goat	4 °C, 120 h, Tris-based medium	Increased	Decreased (CAT, GPx, SOD)	[131]
	Cat	Epididymides stored at 5 °C, 72 h	n.e.	Unchanged (GPx and SOD)	[132]
	Horse	5 °C, 24 h, native and extended (EquiPro) semen	n.e.	Unchanged (CAT, GPx, and SOD) in semen	[143]
Selection	Pig	Discontinuous Percoll gradients	Increased or unchanged (H_2_O_2_)	n.e.	[138]
	Goat	Single layer centrifugation	Increased (O_2_^•−^) or unchanged (H_2_O_2_) in samples selected after FT than in those selected before FT	n.e.	[140]
Sorting	Sheep	Sorting before freezing, Tris-based medium	Decreased (H_2_O_2_) compared to PT unsorted sperm	n.e.	[22]
	Horse	34 °C, 1.5 h, INRA96 modified	Increased	n.e.	[109]
Homogenization	Pig	17 °C, 168 h, none or twice a day homogenization	Unchanged (H_2_O_2_)	Unchanged (TAC, GPx and SOD) or increased (GPx)	[129]
Media composition	Pig	17 °C, 168 h, BTS and Androstar	Unchanged (H_2_O_2_)	Increased (TAC), decreased (SOD), or unchanged (GPx)	[129]
	Cattle	37 °C PT, CPA alone and in combinations	n.e.	Changed depending on CPA (CAT, GPx, GSH)	[134]
	Horse	38 °C, iso- and anisosmotic media	Increased (O_2_^•−^) under anisomotic conditions	n.e.	[135]
Incubation	Cattle	37 °C, 24 h, Andromed extender	Increased (^•^NO, O_2_^•−^, ONOO^−^, ^•^NO_2_, ^•^OH, H_2_O_2_)	n.e.	[113]

* Changes in ROS levels are expressed over total sperm population and compared with fresh semen, unless otherwise specified. BTS: beltsville thawing solution; CAT: catalase; CPA: cryoprotectant; FT: freezing–thawing; GPx: glutathione peroxidase; GSH: glutathione; n.e.: not evaluated; PT: post-thawing; Ref.: references; ROS: reactive oxygen species; SOD: superoxide dismutase; TAC: total antioxidant capacity; TD: time-dependent.
